# Identification of aurora kinase A as an unfavorable prognostic factor and potential treatment target for metastatic gastrointestinal stromal tumors

**DOI:** 10.18632/oncotarget.1705

**Published:** 2014-06-02

**Authors:** Chun-Nan Yeh, Chueh-Chuan Yen, Yen-Yang Chen, Chi-Tung Cheng, Shih-Chiang Huang, Ting-Wei Chang, Fang-Yi Yao, Yung-Chan Lin, Yao-Shan Wen, Kun-Chun Chiang, Jen-Shi Chen, Ta-Sen Yeh, Cheng-Hwai Tzeng, Ta-Chung Chao, Jonathan A. Fletcher

**Affiliations:** ^1^ Department of Surgery, Lin-Kou Medical Center, Chang Gung Memorial Hospital and University, Gueishan Township, Taoyuan County, Taiwan; ^2^ Division of Hematology and Oncology, Department of Medicine, Taipei Veterans General Hospital, Taipei, Taiwan; ^3^ Therapeutical and Research Center of Musculoskeletal Tumor, Taipei Veterans General Hospital, Taipei, Taiwan; ^4^ National Yang-Ming University School of Medicine, Taipei, Taiwan; ^5^ Division of Hematology-Oncology, Department of Internal Medicine, Kaohsiung Chang Gung Memorial Hospital and Chang Gung University College of Medicine, Kaohsiung, Taiwan; ^6^ Department of Pathology, Lin-Kou Medical Center, Chang Gung Memorial Hospital and University, Gueishan Township, Taoyuan County, Taiwan; ^7^ Department of Surgery, Keelung Medical Center, Chang Gung Memorial Hospital and University, Keelung, Taiwan; ^8^ Department of Medical Oncology, Lin-Kou Medical Center, Chang Gung Memorial Hospital and University, Gueishan Township, Taoyuan County, Taiwan; ^9^ Department of Pathology, Brigham and Women's Hospital, Boston, MA, U.S.A

**Keywords:** aurora kinase A, gastrointestinal stromal tumor, imatinib mesylate, MLN8237

## Abstract

Although imatinib mesylate (IM) has revolutionized the management of gastrointestinal stromal tumors (GISTs), drug resistance remains a challenge. Previous studies have shown that the expression of aurora kinase A (AURKA) predicts recurrence in patients with primary, surgically resected GISTs. The current study aimed to evaluate the significance of AURKA expression as an unfavorable prognostic marker for advanced GISTs, and provide evidence that AURKA could be a potential therapeutic target in GISTs. The prognostic significance of the expression of AURKA, along with other clinicopathological factors, was analyzed in a cohort of 99 IM-treated patients with advanced GISTs. The potential use of an inhibitor of AURKA as a therapeutic agent against GISTs was also tested in GIST cell lines. Among 99 enrolled patients, poor performance status, large tumor size, drug response, and AURKA overexpression were independent prognostic factors for poor progression-free survival (PFS). For overall survival (OS), only large tumor size and AURKA overexpression were identified as independent unfavorable factors. In an *in vitro* study, MLN8237, an AURKA inhibitor, inhibited growth of both IM-sensitive and IM-resistant GIST cells in a concentration-dependent manner, and exhibited synergistic cytotoxicity with IM in GIST cells. The inhibitory effect of MLN8237 in GIST cells could be attributed to the induction of G2/M arrest, apoptosis, and senescence. Our study shows that AURKA expression independently predicted poor PFS and OS in patients with advanced GISTs who were treated with IM. An AURKA inhibitor may have potential as a therapeutic agent for both IM-sensitive and IM-resistant GISTs.

## INTRODUCTION

Gastrointestinal stromal tumors (GISTs) are the most common mesenchymal tumors of the gastrointestinal tract. These tumors occur most frequently in the stomach (~50%) and small intestine (25%−35%), and less frequently in the colorectal region (10%−12%), omentum/mesentery (7%), and esophagus (1%−5%) [1–3]. GISTs are thought to originate from the interstitial cells of Cajal (ICC) [4]. The ICC, which are associated with Auerbach's plexus, are innervated cells that have an autonomous pacemaker function and coordinate peristalsis throughout the gastrointestinal tract.

Most GISTs contain mutations in the gene encoding the KIT tyrosine kinase receptor [5], but less than 10% have mutations in the gene encoding the alpha polypeptide of the platelet-derived growth factor receptor (PDGFRA) as the oncogenic driving force [6]. With the development of imatinib mesylate (IM; Novartis Pharmaceuticals, Basel, Switzerland), a potent tyrosine kinase inhibitor (TKI) that inhibits both KIT and PDGFRA, the median overall survival (OS) of patients with advanced GISTs has improved from less than one year in the pre-TKI era to five to six years. However, acquired resistance to IM is inevitable and occurs within two to three years after treatment [7, 8]. The development of resistance is most likely due to resistance-associated mutations [9–11]. For IM-resistant GISTs, the therapeutic options are limited to a few other TKIs such as sunitinib maleate (Pfizer, New York, USA)[12] and regorafenib (BAY 73-4506; Bayer Schering Pharmaceuticals AG, Berlin, Germany) [13]. Thus, there is an urgent need to identify new biomarkers and/or therapeutic targets that can be used to treat these patients.

For localized GISTs, several criteria have been proposed to predict the risk of recurrence. These include the National Institutes of Health (NIH) consensus and modified consensus criteria [14, 15] and the Armed Forces Institute of Pathology (AFIP) criteria [16]. Among them, the mitotic rate is one of the most important risk factors. In a previous study, we re-analyzed available expression profiling data of GISTs and determined that gene sets associated with cell cycle progression or its regulation were strongly associated with the risk of recurrence [17]. Lagarde *et al.* also successfully predicted metastasis in 67 primary untreated GISTs [18] by using a prognostic gene expression signature composed of 67 genes related to chromosome integrity, mitotic control, and genome complexity in sarcomas (Complexity INdex in SARComa, or CINSARC) [19]. Both we and Lagarde *et al.* identified the expression of aurora kinase A (AURKA) as an independent poor prognostic marker for GIST recurrence [17, 18]. However, no data are available regarding the significance of AURKA expression in predicting the prognosis of advanced GISTs. Moreover, it is not clear whether AURKA could be a potential therapeutic target in this type of cancer. This study aimed to address these two issues.

## RESULTS

### High AURKA expression is an independent poor prognostic factor for advanced GISTs

A total of 99 patients with advanced GISTs were enrolled, and their clinicopathological characteristics are summarized in Supplemental Table 1. The mean age of these patients, who were predominantly men, was 57.8 years. Over 80% of the patients had an Eastern Cooperative Oncology Group (ECOG) performance status of 0–1. The small bowel was the most common site (50 of 99; 50.5%), followed by the stomach (37 of 99; 37.4%) and the colon/rectum (8 of 99; 8.1%). The median tumor size (as defined in the PATIENTS AND METHODS section) before treatment with IM was 10.0 cm (range, 2.5–181.0 cm). Genomic analysis was done in 92 cases. Most of the tumors (69.6%) contained mutations in exon 11, some (18.5%) harbored mutations in exon 9, and the remainder (12.0%) were wild type or had mutations in other genes.

**Figure 1:**
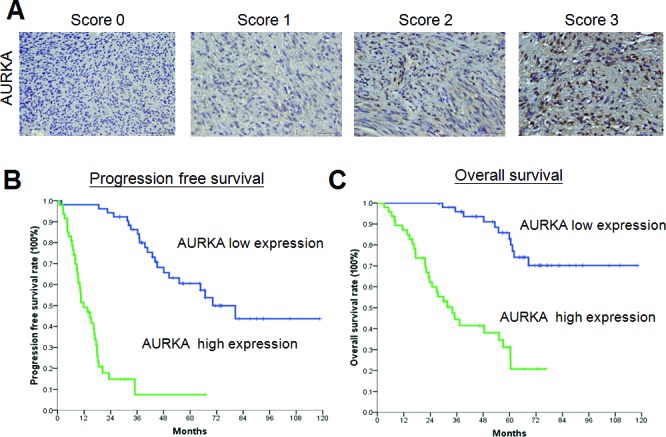
Expression of AURKA in gastrointestinal stromal tumors (GISTs) and the association between AURKA expression and survival (**A**) Representative photomicrographs of samples with low (0%–29% and 30%–59% staining was scored as 0 or 1, respectively) or high (60%–80% and 81%–100% staining was scored as 2 or 3, respectively) expression of AURKA (400x, scale bar = 50 μm). (B-C) Kaplan-Meier plots of (**B**) progression-free survival and (**C**) overall survival of 99 patients with GIST according to AURKA expression. The *P* values for survival comparison, obtained by the log-rank test, were all less than 0.05.

The median follow-up time after IM treatment was 33.6 months (range, 1.6–110.9 months). For all patients, the median progression-free survival (PFS) was 37.6 months and the median OS was 71.0 months. Univariate analysis showed that the PFS of all 99 patients was significantly influenced by age, ECOG performance status, tumor size, platelet count, aspartate aminotransferase (AST) level, AURKA expression level, and treatment response. In the multivariate analysis, however, only high AST level, tumor size greater than 11.5 cm, poor drug response, and AURKA overexpression were identified as independent prognostic factors for poor PFS (Table 1). The Kaplan-Meier PFS curve for AURKA expression is shown in Figure 1B, and those for the other three factors are shown in Supplemental Figure S1.

**Table 1:** Prognostic factors for progression free survival based on univariate analyses and final multivariate model

	Univariate Analysis					Multivariate Analysis		
Factors	Total No.	No. of Events	5-Year PFS (%)	Cumulative Hazard Ratio	Log-Rank *P*		*P*	Hazard Ratio (95% CI)
Age								
≤ 65	71	40	44.3	1				1
> 65	28	19	18.1	2.052	0.01		0.508	1.252 (0.643-2.437)
Sex								
Male	64	38	36.4	1				
Female	35	21	41.4	1.054				
ECOG								
0,1	83	48	41.7	1				1
2,3	16	11	14.6	2.66	0.004		0.309	1.523 (0.677-3.425)
Genetic status								
Exon9	17	12	38.6	1				
Exon11	64	39	36.2	0.786				
Wild type	11	6	36.4	0.902	0.751			
Sum of tumor, cm								
< 11.5	54	28	46.7	1				1
≥ 11.5	45	32	21.5	1.859	0.02		0.047	2.28 (1.19-3.29)
Hemoglobin (g/dL)								
< 12	54	28	27.0	1				
≥ 12	45	32	39.7	0.807	0.429			
HCT (%)								
< 36	55	35	27.2	1				
≥ 36	44	26	36.9	0.887	0.662			
MCV (fL)								
< 80	22	13	27.9	1				
≥ 80	77	49	33.8	1.096	0.787			
Platelet (/μL)								
< 150000	10	9	0	1				
≥ 150000	89	51	37.1	3.371	0.001		0.052	0.352 (0.125-1.02)
Albumin (g/dL)								
< 3.5	34	22	33.5	1				
≥ 3.5	65	37	35.8	0.797	0.469			
INR								
≤ 1.2	78	45	36.2	1				
> 1.2	21	15	31.2	1.268	0.56			
BUN (mg/dL)								
≤ 21	86	52	34.2	1				
> 21	13	9	25.0	1.283	0.542			
Creatinine (mg/dL)								
≤ 1.03	62	38	33.9	1				
> 1.03	37	22	34.7	0.835	0.517			
AST (U/L)								
< 34	80	44	40.5	1				1
≥ 34	19	17	8.3	2.58	0.002		0.032	2.087 (1.067-4.082)
ALT (U/L)								
< 36	83	43	33.7	1				
≥ 36	16	10	23.8	1.511	0.239			
ALK-P (U/L)								
< 94	77	44	36.3	1				
≥ 94	22	17	18.3	1.616	0.138			
Bil (T)								
< 1.0	78	49	31.9	1				
≥ 1.0	21	10	36.6	0.728	0.415			
Sodium (mEq/L)								
< 139	42	27	24.0	1				
≥ 139	57	36	39.0	0.803	0.803			
Potassium (mEq/L)								
≤ 4	38	25	28.0	1				
> 4	61	37	37.4	0.825	0.53			
AURKA								
< 60	52	22	60.5	1				1
> 60	47	37	7.4	10.74	< 0.001		< 0.001	6.567 (2.875-14.999)
Response								
CR/PR	64	31	50.9	1			< 0.001	1
SD	24	17	20.2	2.204			0.045	1.75 (1.016-4.27)
PD	11	11	0.0	NA	< 0.001		< 0.001	180.72 (20.19-1617.31)

Univariate analysis showed that the OS of all 99 patients was also significantly influenced by age, ECOG performance status, tumor size, platelet count, AST level, AURKA expression level, and treatment response in addition to albumin and sodium levels (Table 2). However, only tumor size larger than 11.5 cm and AURKA overexpression were identified as independent unfavorable prognostic factors for OS in the multivariate analysis (Table 2). The Kaplan-Meier OS curve for AURKA expression is shown in Figure 1C and that for tumor size is shown in Figure S2.

**Table 2:** Prognostic factors for overall survival based on univariate analyses and final multivariate model

	Univariate Analysis					Multivariate Analysis		
Factors	Total No.	No. of Events	5-Year OS (%)	Cumulative Hazard Ratio	Log-Rank *P*		*P*	Hazard Ratio (95% CI)
Age								
< 65	71	22	68.1	1				1
> 65	28	19	36.8	3.28	< 0.001		0.118	2.718 (0.775-9.526)
Sex								
Male	64	26	60.4	1				
Female	35	15	57.4	1.126	0.726			
ECOG								
0,1	83	30	67	1				1
2,3	16	11	0	4667	< 0.001		0.082	3.082 (0.866-10.978)
Genetic status								
Exon9	17	7	60.8	1				
Exon11	64	28	59.4	1.24				
Wild type	11	5	51.1	1.349	0.848			
Sum of tumor, cm								
< 11.5	54	18	68.7	1				1
≥ 11.5	45	23	49.2	1.941	0.037		0.039	3.228 (1.14-12.177)
Hemoglobin (g/dL)								
< 12	54	23	52.8	1				
≥ 12	45	19	63	0.752	0.378			
HCT (%)								
< 36	48	23	49.9	1				
≥ 36	51	21	63.2	0.684	0.242			
MCV (fL)								
< 80	22	10	56.2	1				
≥ 80	77	33	57.8	0.90	0.782			
Platelet (/μL)								
< 150000	10	6	32.4	1				1
≥ 150000	89	35	60.3	2.98	0.011		0.695	1.386 (0.271-7.096)
Albumin (g/dL)								
< 3.5	34	19	44.2	1				1
≥ 3.5	65	21	68.1	0.494	0.05		0.094	0.408 (0.143-1.165)
INR								
≤ 1.2	78	27	62.2	1				
> 1.2	21	13	45.5	1.899	0.158			
BUN (mg/dL)								
≤ 21	86	34	60.8	1				
> 21	13	9	35	1.985	0.109			
Creatinine (mg/dL)								
≤ 1.03	62	27	55.9	1				
> 1.03	37	15	61.3	0.954	0.887			
AST (U/L)								
< 34	80	28	65.6	1				1
≥ 34	19	14	31.3	2.58	0.006		0.9	1.073 (0.360-3.196)
ALT (U/L)								
< 36	83	35	59.7	1				
≥ 36	16	8	45.8	1.212	0.648			
ALK-P (U/L)								
< 94	77	30	59.7	1				
≥ 94	22	12	57	1.413	0.374			
Bil (T)								
< 1.0	78	31	61.1	1				
≥ 1.0	21	10	44.5	1.286	0.54			
Sodium (mEq/L)								
< 139	42	25	44.1	1				1
≥ 139	57	18	67.2	0.437	0.02		0.058	0.348 (0.117-1.036)
Potassium (mEq/L)								
≤ 4	38	18	49.3	1				
> 4	61	25	62.3	0.827	0.601			
AURKA								
< 60	52	11	83	1				1
> 60	47	30	31.1	6.748	< 0.001		0.002	9.319(2.320-37.428)
Response								
CR/PR	64	23	63.8	1				1
SD	24	8	71	1.015			0.237	0.394(0.084-1.846)
PD	11	10	13.6	5.95	< 0.0001		0.145	2.748(0.707-10.685)

### MLN8237 inhibits AURKA and induces mitotic arrest in GIST cell lines

**Figure 2:**
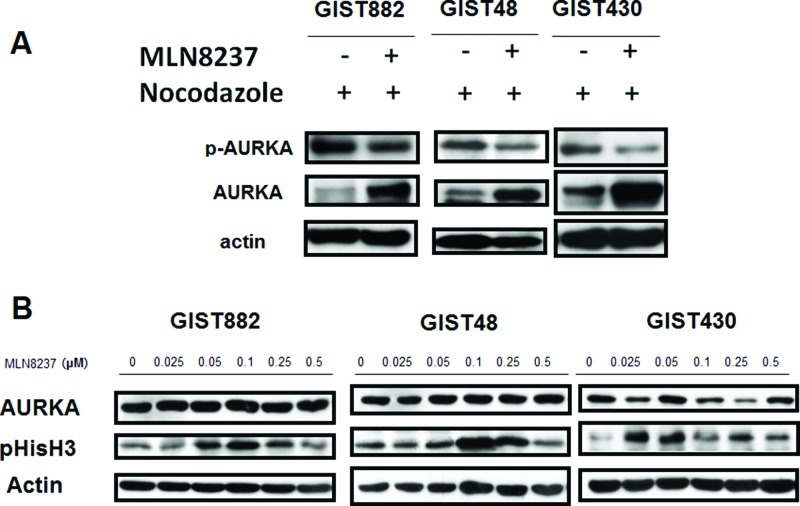
MLN8237 inhibits AURKA and induces mitotic arrest in gastrointestinal stromal tumor (GIST) cell lines **(A)** Immunoblotting with an anti-phospho-Aurora A (Thr288) antibody revealed inhibition of AURKA autophosphorylation in GIST cell lines synchronized by exposure to nocodazole and then treated with MLN8237. **(B)** Immunoblotting of three AURKA-expressing GIST cell lines with an anti-phospho-HistoneH3 (Ser10) (pHisH3) antibody revealed a dose-dependent induction of pHisH3 after treatment with MLN8237 for six days.

We used an *in vitro* model to test the possible use of AURKA as a therapeutic target in GISTs. MLN8237 is a potent inhibitor of AURKA that reduces the activity of AURKA in a variety of cancers [20–22]. We examined whether MLN8237 inhibits the activation of AURKA in GIST cells. Cell division in cultured GIST cells was first synchronized by exposing the cells to nocodazole for 16 hours. The cells were then treated with MLN8237 for 56 hours, after which phosphorylation of AURKA at threonine 288 (pThr288) was measured by western blotting. Decreased phosphorylation of AURKA was detected in all MLN8237-treated GIST cells (Figure 2A). We also demonstrated that all GIST cell lines treated with MLN8237 exhibited a dose-dependent increase in phosphorylation of Histone H3 (phospho-Histone H3; pHisH3) at serine 10 (pSer10), an indicator of mitotic arrest [23](Figure 2B).

### MLN8237 induces G2/M arrest in GIST cell lines with concomitant up-regulation of p21 and/or p53

**Figure 3:**
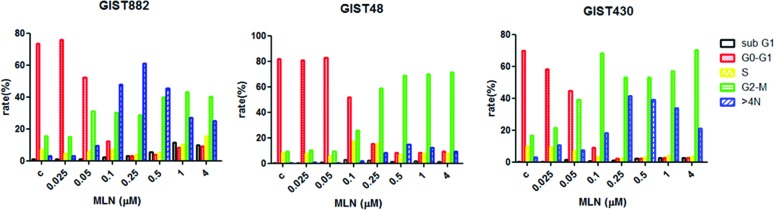
MLN8237 induces G2/M arrest in gastrointestinal stromal tumor (GIST) cell lines The DNA profiles of GIST882, GIST48, and GIST430 cells treated with dimethyl sulphoxide (DMSO) or MLN8237 for six days were evaluated by flow cytometry. The percentages of cells in the sub-G1, G0/G1, S, and G2/M phases, as well as the percentage of cells with a DNA content >4N are shown in different colors.

Because AURKA is a cell cycle regulatory protein, we then explored the effects of MLN8237 on cell cycle progression in GIST cell lines. Flow cytometry analysis of DNA content in cells treated with MLN8237 for six days demonstrated that this compound caused marked accumulation of cells at G2/M as well as >4N DNA content in all three GIST cell lines (Figure 3).

**Figure 4:**
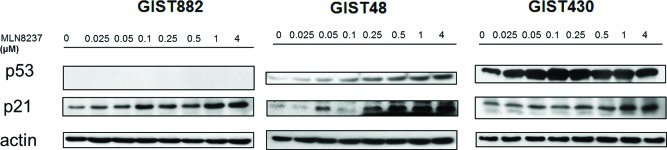
Molecular changes induced by MLN8237 in the p53 pathway were determined by western blotting using gastrointestinal stromal tumor (GIST) cell lines. MLN8237 induced a dose-dependent increase in the expression of p21 in all three cell lines. Dose-dependent up-regulation of p53 could also be seen in GIST48 and GIST430 cells. GIST882 is a p53-negative cell line.

We next analyzed the effect of MLN8237 on the p53 pathway. As shown in Figure 4, treatment with this compound dose-dependently up-regulated the expression of p21 in all three GIST cell lines. In GIST48 and GIST430 cells, there was also a dose-dependent up-regulation of p53 expression. GIST882 is a p53-negative cell line. (Figure 4). These findings indicate that treatment with MLN8237 can increase the expression of p21 and/or p53, which may contribute to cell cycle arrest in GIST cell lines.

### MLN8237 exerts a cytotoxic effect by inducing apoptosis in both IM-sensitive and IM-resistant GIST cell lines and acts synergistically with IM

**Figure 5:**
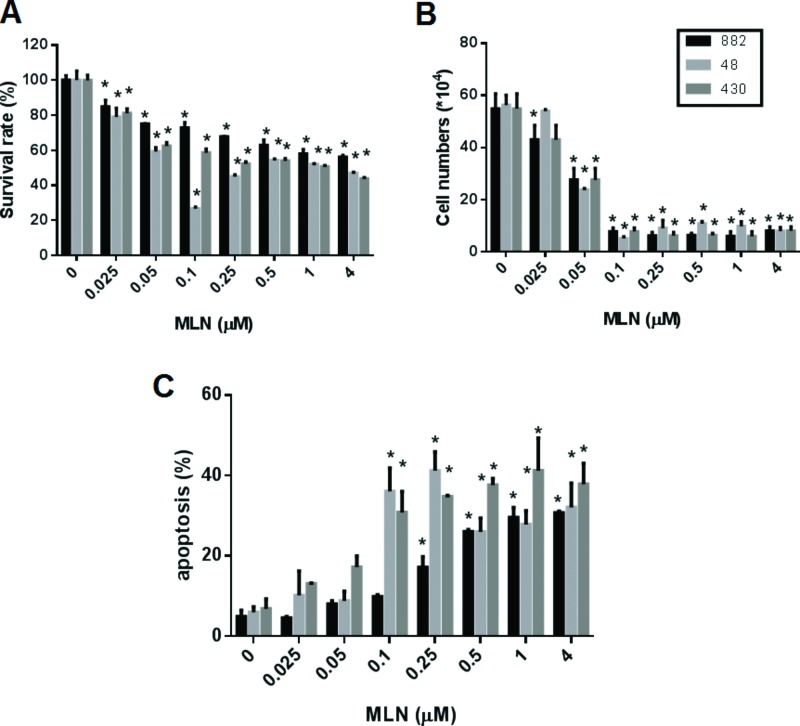
Treatment with MLN8237 inhibits AURKA and results in growth inhibition and apoptosis in gastrointestinal stromal tumor (GIST) cell lines. The viability of GIST882, GIST48, and GIST430 cells after treatment with various concentration of MLN8237 (MLN) for six days was measured with (**A**) the TACS™ MTT cell proliferation assay (expressed as a percentage of viability under controlled culture conditions) and (**B**) a trypan blue exclusion assay (expressed as viable cell number). (**C**) Apoptosis assay. Three GIST cell lines were treated with various concentration of MLN8237 (MLN) for six days. The percentage of apoptotic cells was determined using Annexin V-FITC/propidium iodide (PI) staining. All data represent the mean ± SD of three independent experiments. *P < 0.05.

We examined whether MLN8237 could exhibit cytotoxic activity against GIST cell lines. As shown in Figure 5, MLN8237 displayed cytotoxicity against both IM-sensitive and IM-resistant GIST cells when assessed by an MTT assay (Figure 5A), or by a trypan blue exclusion assay (Figure 5B). A significant induction of apoptosis in GIST cell lines was also detected by co-staining with propidium iodide (PI) and fluorescein isothiocyanate (FITC)-labeled Annexin V (Annexin V-FITC) (Figure 5C).

**Figure 6:**
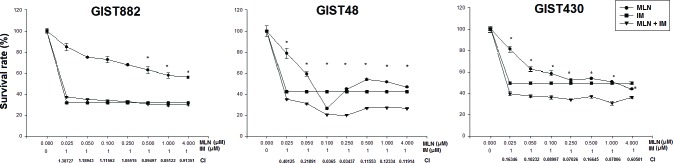
The viability of GIST882, GIST48, and GIST430 cells treated with MLN8237 (MLN) in combination with imatinib (IM) for six days was measured with the TACS™ MTT cell proliferation assay (expressed as a percentage of viability under controlled culture conditions). All data represent the mean ± SD of three independent experiments. *A combination index (CI) < 1.0 indicates a synergistic effect.

We then explored the possible synergistic effects of treatment with both MLN8237 and IM by using a combination index (CI) assay [24]. A CI value <1 is defined as synergy. As shown in Figure 6, treating GIST882 cells with different doses of MLN8237 and a fixed dose of IM revealed that MLN8237 did not synergize with IM except at a relatively high dose. On the other hand, in GIST48 and GIST430 cells, synergy between MLN8237 and IM was observed at all doses tested. These results indicate that MLN8237 can act synergistically with IM in GIST cells, especially in those resistant to IM.

### MLN8237 induces cellular senescence in GIST cell lines

**Figure 7:**
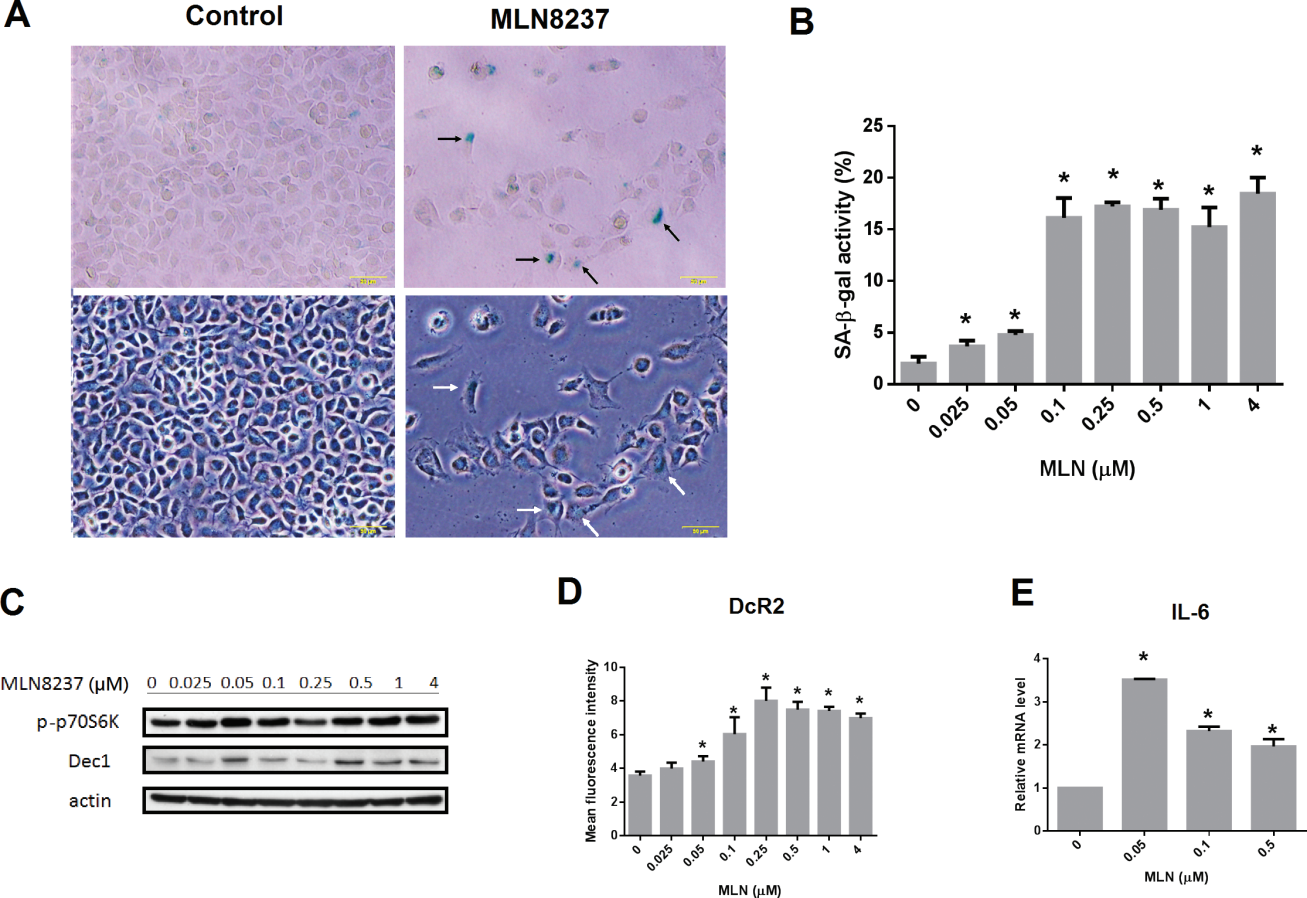
MLN8237 treatment induces cellular senescence in gastrointestinal stromal tumor (GIST) cell lines (**A**) Representative photomicrographs of GIST48 cells before and after MLN8237 treatment. Upper panel: images of cells after staining for senescence-associated β-galactosidase (SA-β-gal) activity; lower panel: corresponding bright field images. Scale bar = 50 μm. Some SA-β-gal positive cells are indicated by arrows. (**B**) Treating GIST48 cells with MLN8237 for six days dose-dependently increased SA-β-gal staining. (**C**) Western blot analysis of phospho-p70 S6 kinase (p-p70S6K) and DEC1 expression relative to that of actin in GIST48 cells treated with MLN8237. (**D**) Flow cytometric analysis of the expression of DcR2 in GIST48 cells treated with MLN8237 for six days. (**E**) Quantitative reverse transcription-polymerase chain reaction (qRT-PCR) analysis of interleukin-6 (IL-6) expression relative to that of glyceraldehyde 3-phosphate dehydrogenase (GAPDH) in GIST48 cells treated with MLN8237. ^*^P < 0.05.

Inhibition of AURKA may induce senescence in cells. To examine whether senescence occurred in GIST cells following treatment with MLN8237, a senescence-associated β-galactosidase (SA-β-gal) assay was performed in GIST48 cells after being treated with MLN8237 for six days. MLN8237 treatment significantly increased SA-β-gal activity in GIST cells (Figure 7A and 7B). Furthermore, administration of MLN8237 also increased the expression of DEC1 (Figure 7C) and DcR2 (Figure 7D), two well-known senescence biomarkers [25, 26], in a dose-dependent manner, while the levels of phospho-p70 S6 kinase remained steady. As measured by quantitative reverse transcription-polymerase chain reaction (qRT-PCR), the expression of interleukin 6 (IL-6), a cytokine associated with the senescence-associated secretory phenotype (SASP) [25, 26], was also up-regulated in GIST48 cells treated with MLN8237 (Figure 7E). Collectively, these results demonstrate that MLN8237 treatment induces senescence in GIST cells.

## DISCUSSION

In this study, we found that overexpression of AURKA was an independent poor prognostic factor for both PFS and OS among 99 patients with advanced GISTs who were treated with IM. In *in vitro* assays, the AURKA inhibitor MLN8237 inhibited the growth of both IM-sensitive and IM-resistant GIST cells in a concentration-dependent manner and exerted synergistic cytotoxicity with IM. The inhibitory effect of MLN8237 on GIST cells could be attributed to the induction of G2/M arrest, apoptosis, and senescence.

In the past few years, several studies have shown that genes involved in cell cycle regulation are important in the pathogenesis of sarcoma. Chibon *et al*. have established that CINSARC, a gene expression signature composed of 67 genes related to chromosome integrity, mitotic control, and genome complexity, could predict metastasis outcome in sarcoma [19]. Moreover, by comparing transcriptional profiling data of uterine myometrium, leiomyoma, and leiomyosarcoma (ULMS), Shan *et al.* discovered that 26 of the 50-most overexpressed genes in ULMS regulate mitotic centrosome and spindle functions [27]. In GISTs, both our group (by re-analyzing available expression profiling data of GISTs) [17] and Lagarde *et al*. (by using the CINSARC signature) [18] have determined that genes involved in the progression or regulation of the cell cycle were strongly associated with the risk of GISTs recurrence. Most interestingly, the findings of Shan *et al*., our group, and Lagarde *et al*. highlight the important role of AURKA in the pathogenesis of ULMS and GISTs [17, 18, 27].

AURKA plays a key role in the regulation of cell cycle progression [28]. This protein is overexpressed in a wide range of tumors and is considered an unfavorable prognostic factor [29–32]. In addition, the ability of AURKA to transform has also been shown in several types of cells [33]. Most importantly, several inhibitors of AURKA are currently being tested in clinical-phase studies [34]. Therefore, we investigated the potential of AURKA inhibitors for use as therapeutic agents in the treatment of GISTs.

MLN8237 is an AURKA inhibitor whose anti-tumor activity has been confirmed in a variety of cancers [20–22, 35]; furthermore, it has been tested in phase I clinical trials [36, 37]. In this study, we demonstrated that MLN8237 inhibited activation of AURKA (Figure 2A) with resultant mitotic inhibition, as reflected by increased expression of pHisH3 [23] (Figure 2B) in all three cell lines tested. In addition, we found that incubating GIST cells with MLN8237 consistently induced G2/M cell cycle arrest (Figure 3). Our study also revealed the ability of MLN8237 to inhibit cell growth and proliferation, as well as induce apoptosis, in both IM-sensitive and IM-resistant GIST cells (Figure 5). Finally, MLN8237 exerted synergistic cytotoxicity with IM in GIST cells (Figure 6). These findings are similar to those of investigators studying other types of tumors [20–22]. It is interesting to note that the cytotoxicity of MLN8237 could only be detected after six days of treatment. One possible explanation for this finding is that the doubling time of GIST cell lines is relatively longer than that of other sarcoma cell lines. It is also interesting to note that MLN8237 treatment up-regulated the expression of p21 (Figure 4). p21 has been shown to play a crucial role in G1 and G2 checkpoint control [38], and its role in MLN8237-induced cell cycle arrest in GIST cells deserves further investigation.

Furthermore, our studies determined that treatment with MLN8237 induced cellular senescence in GIST cells (Figure 7). Cell cycle arrest is not sufficient to cause senescence. MLN8237 treatment inhibits AURKA, thus causing cell cycle arrest (quiescence), but leaves the proliferative signaling pathway (mTOR) intact (Figure 7C), which could converts an arrest into senescence (geroconversion) [39]. In this study, we demonstrated that MLN8237 not only significantly increased SA- ß-gal activity in GIST cells (Figure 7A and 7B), but also induced expression of senescence biomarkers such as DEC1, DcR2, and IL-6 (Figure 7C, 7D, and 7E) [25, 26]. Cellular senescence is an important phenotype resulting from treatment with cell cycle inhibitors [40, 41], and senescence-associated markers may become important biomarkers for therapy with these compounds. The role of senescence in the cytotoxic mechanism of these agents awaits further clarification.

Several clinical factors *e.g.*, age, performance status, tumor size, and treatment response, were also identified as prognostic factors among patients with advanced GISTs. Among them, tumor size clearly was one of the most important factors in predicting PFS and OS. In this study, the size of the five largest lesions was measured and defined by the RECIST criteria [42], and the sum of the largest dimension was used as tumor size. Tumor size is known to be associated with recurrence of localized GISTs [14–16] and to be a prognostic factor for OS of patients with advanced GISTs [7, 8, 10]. Treatment response was also an independent prognostic factor for PFS, as expected [7, 8].

In the MetaGIST study, several laboratory data were shown to have prognostic significance, *e.g.*, abnormal blood count and albumin level [10]. In this study, platelet count and albumin level had prognostic significance in predicting either OS or PFS by univariate analysis. However, neither was an independent factor in multivariate analysis. Interestingly, an elevated AST level was an independent prognostic factor for OS; however, the reason for this association remains unknown. The mutation status of a patient failed to be a prognostic factor, probably because of the limited number of samples tested.

In conclusion, the current study showed that the expression of AURKA was an independent poor prognostic factor for both PFS and OS in patients with advanced GISTs who were treated with IM. Inhibition of AURKA with MLN8237 may represent a novel therapeutic strategy for the treatment of advanced GISTs.

### Statement of translational relevance

Our previous studies revealed that AURKA overexpression predicts recurrence in patients with primary, surgically resected, gastrointestinal stromal tumors (GISTs). In the current study, we determined that overexpression of AURKA is also an independent poor prognostic factor of both PFS and OS in patients with advanced GISTs. By using an *in vitro* model, we also determined that MLN8237, an AURKA inhibitor, inhibits growth of both IM-sensitive and IM-resistant GIST cells and exerts synergistic cytotoxicity with IM. The mechanism of inhibition could be attributed to the induction of G2/M arrest, apoptosis, and senescence. The role of AURKA as a potential therapeutic target for GISTs should be further explored in *in vivo* models as well as in clinical trials. Cellular senescence is an important phenotype resulting from treatment with cell cycle inhibitors, and senescence-associated markers may become important biomarkers for therapy with these compounds.

## PATIENTS AND METHODS

### Analysis of 99 patients with metastatic GISTs who received imatinib

The study protocol for the collection of tumor samples and clinical information was approved by the institutional review board, and patients provided written informed consent authorizing the collection and use of their tumor samples for research purposes. Only patients with histologically confirmed metastatic GISTs that expressed the CD117 antigen were eligible. All patients were treated with 400 mg of IM as first-line therapy. Standard computed tomography (CT) imaging was performed on each patient every three months for the first three years and every six months for the following two years to assess the patient's response. Tumor size was measured in at least five target lesions; the sum of the largest dimension was used as an initial size measurement as well as a response evaluation indicator, as recommended by RECIST [42].

### Immunohistochemical analysis of AURKA expression in metastatic GISTs

To determine the expression of AURKA, a 4-μm section of each specimen was subjected to immunohistochemical analysis. The primary antibody against AURKA (rabbit anti-Aurora A polyclonal antibody; NOVUS NB100-212) was diluted (1:1500) and applied to the slides, which were then incubated overnight at 4°C. The slides were then washed three times for five minutes in TBST before visualization with the DAKO LSAB2 System-HRP (No K0675, DAKO A/S, Denmark). Control slides were incubated with secondary antibody only. After washing three times for five minutes in TBST, the slides were mounted. We used microscopy to analyze the slides in a blinded fashion. The expression of AURKA in GISTs was scored as either low (0%−29% and 30%−59% staining was scored as 0 or 1, respectively) or high (60%−80% and 81%−100% staining was scored as 2 or 3, respectively) (Figure 1A).

### Statistical and survival analysis

PFS was defined as the length of time with no progression after administration of IM. OS was defined as survival after the administration of IM. All patients were followed until death or December 2010. The correlations between clinicopathological variables and AURKA expression were analyzed by the χ2 or Fisher's exact tests. Survival was estimated with the Kaplan-Meier method and the log-rank test was used to compare the survival curves. Univariate and multivariate (stepwise forward conditional method) Cox regression analyses were used to determine the prognostic significance of clinicopathological factors and AURKA expression. A *P* value of <0.05 was regarded as statistically significant in two-sided tests. The SPSS software (version 13.00, SPSS, Chicago, IL, USA) was used for all statistical analyses.

### Cell lines and reagents

All three GIST cell lines (GIST882, GIST48, and GIST430) were kindly provided by Dr. JA Fletcher. The mutation status of the KIT gene in these cell lines has been previously described [43]. GIST882 is an IM-sensitive cell line with a homozygous missense mutation in exon 13 of KIT (K642E) [44]. The GIST430 line, which harbors a primary, heterozygous, inframe deletion in exon 11 and a secondary, heterozygous, missense mutation in exon 13 in KIT, and the GIST48 line, which harbors a primary, heterozygous, missense mutation in exon 11 and a secondary, heterozygous, missense mutation in exon 17 in KIT, are both relatively IM-resistant [43]. MLN8237, an AURKA-selective inhibitor, was purchased from Selleck Chemicals. It was dissolved in dimethyl sulfoxide (DMSO) to a stock concentration of 10 mM. Nocodazole was purchased from Sigma-Aldrich (CAS Number 31430-18-9). The following antibodies were used for immunoblotting: anti-Aurora A/AIK (#3092; 1:1000), anti-phospho-Aurora A (Thr288) (#3079; 1:500), anti-phospho-HistoneH3 (Ser10) (pHisH3) (#9701; 1:1000), anti-p53 (#2524; 1:1000), and anti-phospho-p70 S6 Kinase (#9205; 1:500), all from Cell Signaling Technology; anti-p21 (sc-817; 1:1000) and anti-DEC1 (sc-101023; 1:500), both from Santa Cruz Biotechnology; and anti-actin (ABS 24-100; 1:50000).

### Western blotting

Monolayers of cultured cells were rinsed with phosphate-buffered saline (PBS) and scraped into lysis buffer (25 mM TrisHCl, pH 7.6, 150 mM NaCl, 1% NP-40, 1% sodium deoxycholate, and 0.1% SDS [Thermo]) containing a protease and phosphatase inhibitor cocktail (1:100 dilution; Thermo). The lysates were incubated for 30 minutes at 4°C and then clarified by centrifugation for 30 minutes at 13200 r.p.m. at 4°C. The protein concentrations of the supernatants were determined with the Pierce BCA Protein Assay Kit (Thermo). Protein extracts (20-50 μg per lane) were electrophoretically separated on sodium dodecyl sulfate–polyacrylamide gels, transferred to polyvinylidene fluoride membranes (Millipore), and incubated with specific antibodies. The immunoreactive bands were detected with an enhanced chemiluminescence system (Millipore) and X-ray films.

### Cell cycle analysis

Cell cycle analysis was performed by flow cytometry, as previously described [45]. Briefly, cells were trypsinized, washed twice with PBS, and fixed in 70% ethanol at -20°C for two hours. The fixed cells were then washed twice with cold PBS and suspended in 420 μl of PBS. Next, 50 μl of 10 mg/ml RNase A (Sigma) was added and the samples were incubated at 37°C for 30 minutes. Then 20 μl of 0.2 mg/ml PI was added and the cells were incubated at room temperature for 10 minutes. Flow cytometry was performed on a FACS Calibur (Becton Dickinson and Co., Oxford, CA, USA) and the relative DNA content was determined on the basis of the intensity of the red fluorescence. The percentage of cells in each phase of the mitotic cell cycle was calculated with the CellQuest software (Becton Dickinson & Co.).

### 3-(4,5-Dimethylthiazol-2-yl)-2,5-diphenyltetrazolium bromide (MTT) assay

The viability of the cells was measured with the TACS™ MTT cell proliferation assay (TREVIGEN systems) in accordance with the manufacturer's instructions. Briefly, cells were plated in 96-well plates at a concentration of 2000-20000 cells/100 μl/well. The next day, drugs were added at different concentrations; all experiments were performed in triplicate. The plates were incubated for six days at 37°C. Then, 10 μl of the MTT solution was added to each well and the plates were incubated for an additional four hours at 37°C. Then, a detergent solution (200 μl/well) was added and mixed thoroughly to dissolve the dark-blue crystals. The absorbance of the converted dye was measured spectrophotometrically with a microplate reader (Vmax, Molecular Devices, Sunnyvale, CA, USA) at 570 nm (test) and 650 nm (reference). Cell survival was calculated as the percentage of MTT inhibition as follows: % survival = (mean experimental absorbance/mean control absorbance) × 100 [46].

The possible synergistic effect of MLN8237 and IM was analyzed with the CalcuSyn software program (Biosoft, Ferguson, MO, USA), which is based on the Chou and Talalay method [24]. The combination index (CI) was calculated with CalcuSyn software. For the CI, a value >1 is defined as antagonism, equal to 1 as additivity, and <1 as synergy. The experiment was performed in triplicate.

### Trypan blue exclusion assay

The viability of the cells was also measured with a trypan blue exclusion assay as previously described [47]. GIST cells were seeded at 5 × 10^4^ cells/well in 24-well plates. The next day, a variable concentration of MLN8237 was added to each well and the plates were incubated at 37°C for six days. All experiments were performed in triplicate. Then the cells were trypsinized and mixed with trypan blue. Viable cells have intact cell membranes; thus, trypan blue is not absorbed, but dead cells do absorb it. We counted the number of viable cells with a light microscope.

### Detection of apoptosis

Drug-induced apoptosis was measured with the Annexin V-FITC Apoptosis Detection Kit (BD Pharmingen, San Diego, CA, USA). After treatment with MLN8237, the cells were washed once with 1x PBS and resuspended in 100 μl of staining solution (containing Annexin V-FITC and PI in a HEPES buffer). After being incubated at room temperature for 15 minutes, the cells were diluted in 1x Annexin V–binding buffer and the percentage of apoptotic cells was determined by flow cytometry FACS Calibur (Becton Dickinson and Co., Oxford, CA, USA) [48].

### SA-β-gal assay

SA-β-gal activity was detected with the Cellular Senescence Assay Kit (Millipore) as described in the manufacturer's instructions. GIST48 cells were treated with MLN8237 for six days. The adherent cells were fixed and stained with X-gal in a staining solution at pH 6.0. The cells were washed twice with 1x PBS. The percentage of SA-β-gal-positive cells (the number of positive cells relative to the total number of cells) was quantified by counting 100 cells in three randomly chosen fields per dish with an OLYMPUS IX51.

### Analysis of DcR2 expression by flow cytometry

Flow cytometry was used to detect the expression of DcR2. After MLN8237 treatment, the cells were washed twice with 1x PBS and then incubated with Alexa Fluor 488-labeled anti-DcR2 (R&D Systems, Minneapolis, MN, USA) for 30 minutes on a shaker at room temperature. The cells were washed twice with 1x PBS and then resuspended in 1x PBS. The mean of the fluorescence intensity on the cell surface was determined by flow cytometry FACS Calibur (Becton Dickinson and Co., Oxford, CA, USA).

### Analysis of IL-6 expression with qRT-PCR

Total RNAs were isolated from the GIST 48 cells with TRIzol® Reagent (Invitrogen) in accordance with the manufacturer's instructions. Reverse transcription was performed with 1 μg RNA with the SuperScript^®^ III First-Strand Synthesis System for RT-PCR (Invitrogen). The mRNA copy number for both IL-6 and glyceraldehyde 3-phosphate dehydrogenase (GAPDH) was determined by qRT-PCR with Maxima SYBR Green/ROX qPCR Master Mix (Thermo Scientific) and a LightCycler^®^ 480 System (Roche). The primer sequences used in the qRT-PCRs were as follows: IL-6 (F), 5' CATTTGTGGTTGGGTCAGG 3'; IL-6 ^®^, 5' AGTGAGGAACAAGCCAGAGC 3'; GAPDH (F) 5', GCCAAGGTCATCCATGACAACT 3'; GAPDH ^®^, 5' GAGGGGCCATCCACAGTCTT 3' [26]. The cycling conditions were as follows: 95°C for five minutes and then 45 cycles of 95°C for 30 seconds, 55°C for 30 seconds, and 72°C for 40 seconds. The gene expression levels were calculated as described previously [49].

## SUPPLEMENTARY FIGURES AND TABLE


