# Supplementary material for: Identification of aurora kinase A as an unfavorable prognostic factor and potential treatment target for metastatic gastrointestinal stromal tumors
Source: Oncotarget. 2014 Jun 2;5(12):4071–86. doi: 10.18632/oncotarget.1705 (PMC4147307; doi:10.18632/oncotarget.1705)
Supplement: Supplementary file 1 [file oncotarget-05-4071-s001.pdf]

SUPPLEMENTARY FIGURES AND TABLE

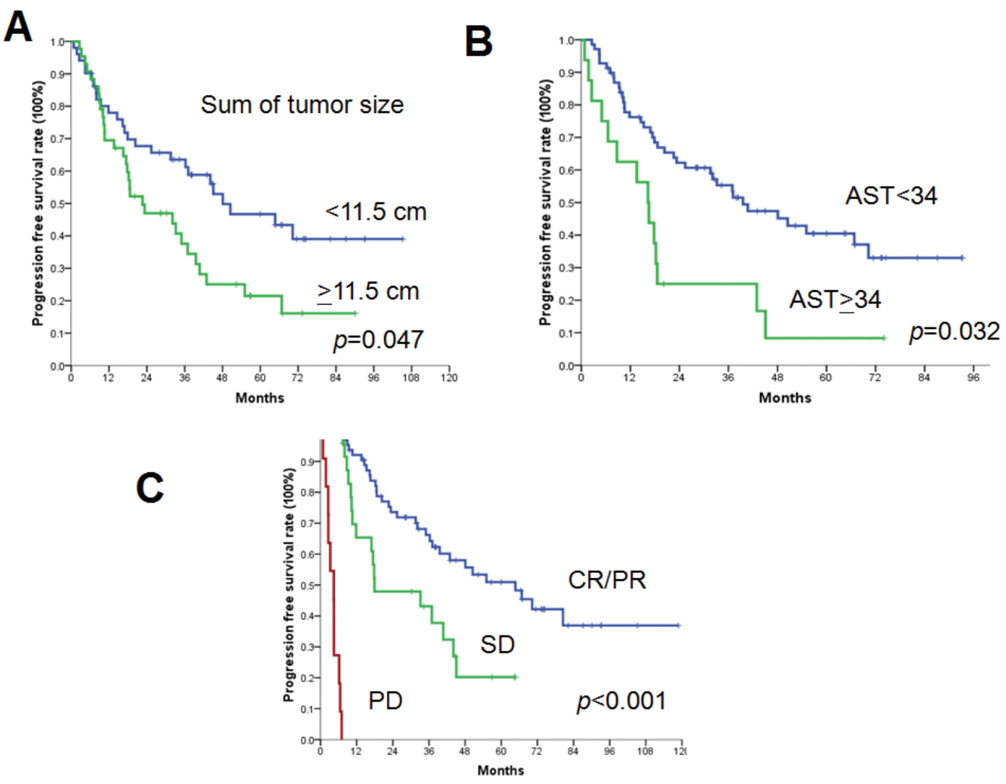

Figure S1: Kaplan-Meier plot of progression-free survival of 99 gastrointestinal stromal tumors (GISTs) patients according to **(A)** sum of tumor size **(B)** AST level and **(C)** treatment response. The P values for survival comparison, obtained by the log-rank test, were all less than 0.05.

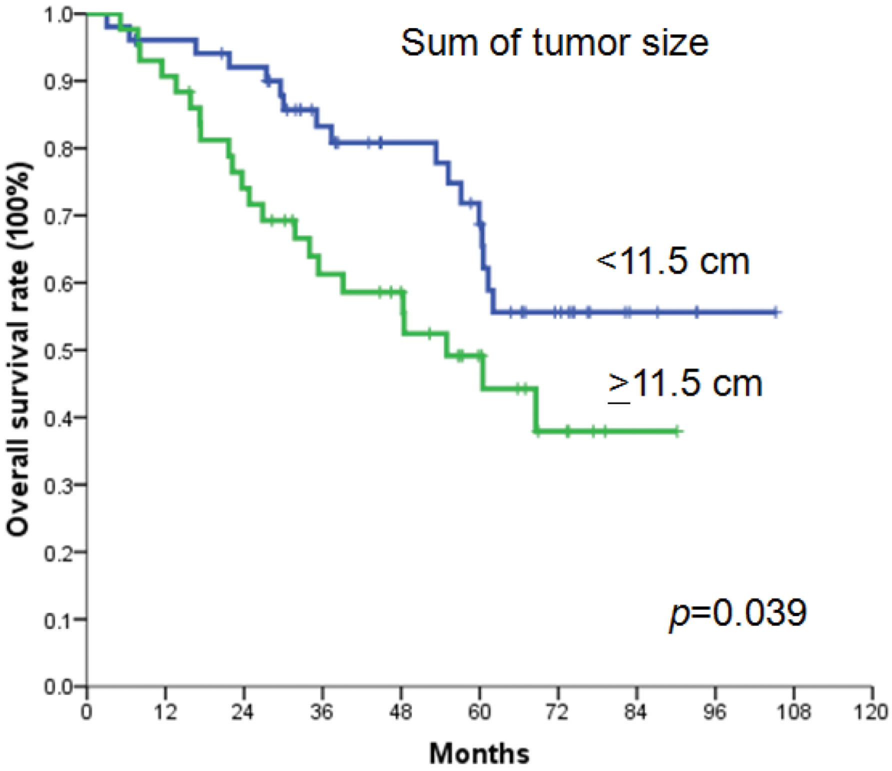

Figure S2: Kaplan-Meier plot of overall survival of 99 gastrointestinal stromal tumors (GISTs) patients according to sum of tumor size. The P value for survival comparison, obtained by the log-rank test, was less than 0.05.

Table S1: Demographic data of the 99 Taiwan GIST patients receiving imatinib mesylate

Patients' characteristics	NO.(%)
Age (median/range)(years)	57.8 (29-82)
Gender (Male: Female)	64:35
ECOG	
0	43 (43.4%)
1	40 (40.4%)
2	14 (14.1%)
3	2 (2.1%)
Origin of GIST	
Stomach	37 (37.4%)
Small bowel	50 (50.5%)
Colon/rectum	8 (8.1%)
Others	4 (4.0%)
Tumor size before imatinib treatment (median/range; cm)	10 (2.5-181.0)
Genetic spectrum of 92 patients tested	
Exon 11 mutation	64 (69.6%)
Exon 9 mutation	17 (18.5%)
Wild type and others	11 (12.0%)
